# *Burkholderia* and *Paraburkholderia* are Predominant Soybean Rhizobial Genera in Venezuelan Soils in Different Climatic and Topographical Regions

**DOI:** 10.1264/jsme2.ME18076

**Published:** 2019-02-15

**Authors:** María Daniela Artigas Ramírez, Mingrelia España, Claudia Aguirre, Katsuhiro Kojima, Naoko Ohkama-Ohtsu, Hitoshi Sekimoto, Tadashi Yokoyama

**Affiliations:** 1 United Graduate School of Agriculture, Tokyo University of Agriculture and Technology (TUAT) Saiwai-cho 3–5–8, Fuchu, Tokyo 183–8509 Japan; 2 Institute for Advanced Studies (IDEA) Miranda Venezuela; 3 National Laboratory of Biofertilizer (INSAI) Aragua Venezuela; 4 Faculty of Agriculture, Tokyo University of Agriculture and Technology 183–8509 Japan; 5 Institute of Agriculture, Tokyo University of Agriculture and Technology (TUAT) Saiwai-cho 3–5–8, Fuchu, Tokyo 183–8509 Japan; 6 Faculty of Agriculture, Utsunomiya University Utsunomiya 321–8505 Japan

**Keywords:** soybean rhizobia, Venezuela, *Rhizobium*, *Bradyrhizobium*, *Burkholderia*

## Abstract

The climate, topography, fauna, and flora of Venezuela are highly diverse. However, limited information is currently available on the characterization of soybean rhizobia in Venezuela. To clarify the physiological and genetic diversities of soybean rhizobia in Venezuela, soybean root nodules were collected from 11 soil types located in different topographical regions. A total of 395 root nodules were collected and 120 isolates were obtained. All isolates were classified in terms of stress tolerance under different concentrations of NaCl and Al^3+^. The tolerance levels of isolates to NaCl and Al^3+^ varied. Based on sampling origins and stress tolerance levels, 44 isolates were selected for further characterization. An inoculation test indicated that all isolates showed the capacity for root nodulation on soybean. Based on multilocus sequence typing (MLST), 20 isolates were classified into the genera *Rhizobium* and *Bradyrhizobium*. The remaining 24 isolates were classified into the genus *Burkholderia* or *Paraburkholderia*. There is currently no evidence to demonstrate that the genera *Burkholderia* and *Paraburkholderia* are the predominant soybean rhizobia in agricultural fields. Of the 24 isolates classified in (*Para*) *Burkholderia*, the *nodD–nodB* intergenic spacer regions of 10 isolates and the *nifH* gene sequences of 17 isolates were closely related to the genera *Rhizobium* and *Bradyrhizobium*, respectively. The root nodulation numbers of five (*Para*) *Burkholderia* isolates were higher than those of the 20 α-rhizobia. Furthermore, among the 44 isolates tested, one *Paraburkholderia* isolate exhibited the highest nitrogen-fixation activity in root nodules.

Venezuela lies within the tropics and its climate ranges from tropical humid to alpine. The country mainly falls into four horizontal temperature zones, depending on elevation and topography: tropical, temperate, cool, and permanent snowfields. Based on the United States Department of Agriculture (USDA) soil taxonomy, nine soil types are recognized in Venezuela (10, 54, 57). Some soil properties restrict agricultural practices, such as acidic soil with high aluminum (Al) availability, which is distributed among Latin-American countries, including Venezuela (21, 26, 42). Approximately 70% of the arable land in Venezuela has low soil pH values. These soils are also less fertile because of nitrogen and phosphorus deficiencies (10, 42, 54). However, variations in topography, geology, and climate at the locations of the different soil types, which are distributed throughout Venezuela, have led to extraordinarily diverse flora and fauna, particularly microorganisms associated with plants (57). Thus, diverse agricultural products have evolved through adaptations to the various ecosystems (1). However, for continued improvements in agricultural production in terms of crop yield, ecosystem functioning, including soil–microorganism–plant interactions, needs to be more extensively examined.

Soil bacteria collectively referred to as rhizobia induce the formation of nodules on the roots of host legumes. The genera *Rhizobium*, *Bradyrhizobium*, *Sinorhizobium* (*Ensifer*), *Mesorhizobium*, *Azorhizobium*, and *Allorhizobium*, which belong to the α-proteobacteria group (α-rhizobia), have been studied in detail as root nodule bacteria (20, 60, 75). Several isolates of the genera *Methylobacterium*, *Devosia*, and *Frankia* have been classified as root nodule bacteria (69, 75). Root nodule bacteria have also been discovered in a class of β-proteobacteria (β-rhizobia). Most *Burkholderia* species have been isolated from host plants classified in the subfamily *Mimosoideae*. *Burkholderia* species isolated from *Papilionoideae* species are distributed in tropical regions of Africa and South America (51), and isolates of *Cupriavidus* (formerly *Ralstonia*) have also been identified as root nodule bacteria (2, 12, 13, 75). These findings support the theory of symbiotic lateral gene transfer from rhizobia to other bacteria (17, 18, 49, 59, 75). Chen *et al*. (13) also reported that *Cupriavidus taiwanensis* was the predominant symbiont of *Mimosa pudica* and *M. diplotricha* in Taiwan, and several isolates of *Burkholderia phymatum* and other species were confirmed as root nodule symbionts of *Mimosa* species (12, 21, 50, 75).

In *Rhizobium*–legume symbiosis, the number of root nodules was found to strongly correlate with the physiological state of the host plant (15, 20, 55) as well as environmental conditions, such as inoculation histories, land-use patterns, and soil properties (53, 65, 74). Consequently, populations of rhizobial species vary according to their abilities to adapt to the environment (30, 52, 75). For example, some fast-growing rhizobia isolated from dry areas of Syria, Lebanon, and Morocco exhibited higher tolerance to NaCl concentrations greater than 34 mM than slow-growing rhizobia such as *Bradyrhizobium japonicum* strains (18, 24, 64). Indrasumunar *et al*. (33) confirmed that the application of acid-tolerant rhizobial species isolated from soybean root nodules grown in acidic soils is needed for soybean cultivation in acidic soils.

In 1971, Barrios and Gonzalez (7) surveyed the root nodules of indigenous legumes growing in Venezuelan savannas, and confirmed root nodulation on 127 species, which accounts for 13% of all *Leguminosae* in Venezuela. Marquina *et al*. (45) reported data on salinity resistance, pH, temperature growth conditions, and intrinsic antibiotic resistance for 12 Venezuelan rhizobial isolates. Soybean is an important leguminous crop worldwide and was recently introduced into Venezuelan agriculture. However, no information is currently available on soybean–rhizobium symbiosis under Venezuelan environmental conditions. The present study is the first to investigate root nodule bacteria for soybean in Venezuela. We isolated several root nodule bacterial species from soybean root nodules in Venezuela and characterized these isolates based on physiological and molecular biological analyses. The results obtained demonstrated that *Burkholderia* are the predominant soybean root-nodulating bacteria in Venezuelan soils located in different climatic and topographical regions.

## Materials and Methods

### Soil samples and collection sites

**Fig. 1:**
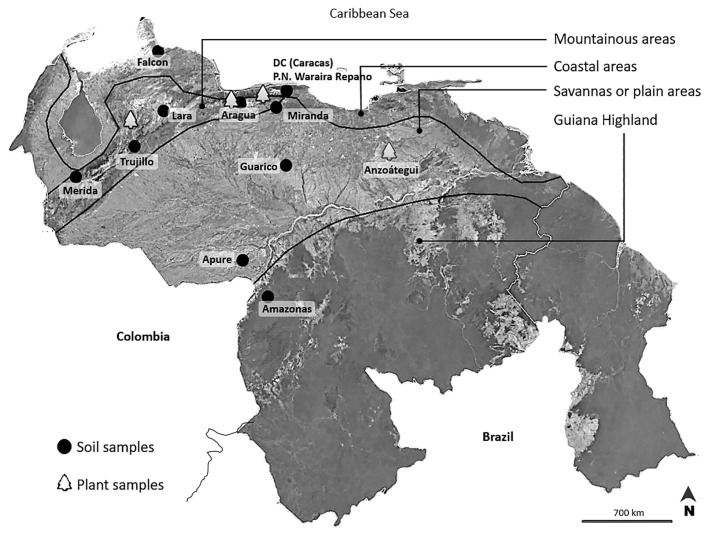
Map of Venezuela showing different agro-ecological regions as collection sites, and geographical locations of soil and plant samples used for rhizobial isolation.

Soil samples representing different soil types, such as Alfisol, Oxisol, Inceptisol, Aridisol, Ultisol, and Vertisol, were collected from 11 areas in Venezuela (Fig. 1, Table 1). The field collection of root nodules was conducted in three areas, as listed in Table 1. These areas were located in diverse agro-ecological regions with contrasting climates, topographies, and soils (Table 1). Soil samples from each area were a composite of two subsamples prepared by mixing soils obtained from a depth of 0–20 cm. No bacterial inoculations had previously been performed in these areas; therefore, the isolates were considered to be indigenous to Venezuela.

### Isolation of rhizobia from Venezuelan soils using soybean cultivars as trap hosts

Soybean seeds of *Glycine max* ‘INIA’ (a Venezuelan cultivar) and ‘Enrei’ (a Japanese cultivar) were surface-sterilized and inoculated with five-fold dilutions of soil suspensions as described previously (22, 60). After sowing the seeds in 300-mL glass jars containing sterilized vermiculite, the jars were transferred to a growth chamber room. Sterilized N_2_-free nutrient solution (65) was added to vermiculite in the jar to a moisture content of 60%, which was maintained throughout the growth period. Plants were grown for 4 weeks in the growth chamber at 28°C under a 16-h light/8-h dark photoperiod. After 4 weeks, the root nodules were harvested and surface-sterilized using 70% (v/v) ethanol for 1 min and 3% (v/v) sodium hypochlorite for 2 min, then rinsed four times with sterile distilled water. Sterilized root nodules were crushed in 300 μL glycerol solution (15% [v/v]) to obtain a turbid suspension. An aliquot (10 μL) of the suspension was streaked (74) onto 1.5% (w/v) yeast extract mannitol agar (YMA) (65) and incubated at 28°C for 1 week. The remaining suspension was frozen at −80°C for further isolation. Single colonies were picked up and then re-streaked onto fresh plates to obtain pure colonies for further analyses. The isolates tested were phenotypically characterized in terms of their growth rate (on plates and broth), texture, and color on YMA (65). Isolates were further tested to evaluate their nodulation ability and symbiotic performance.

### Stress tolerance screening

To assess the tolerance of isolates to different NaCl concentrations, 5 μL of the culture solutions (10^9^ cells mL^−1^) of isolates was incubated on YMA supplemented with 0 (the control), 1, 2, 3, or 4% (w/v) NaCl (Kanto Chemical, Tokyo, Japan). Plates were incubated at 28°C for 4–5 d (22), and the growth rate of each colony was scored as follows: no growth (−), 10–20% more growth than the control (+), 30–60% more growth than the control (++), and 70–100% more growth than the control (+++).

To assess the Al tolerance of isolates, 5 μL of the culture solution (10^9^ cells mL^−1^) of isolates was incubated on YMA supplemented with AlCl_3_ (Wako Pure Chemical Industries, Osaka, Japan) concentrations of 0.1 (as the control), 0.5, 1, or 2 mM in combination with acidic (pH 4.5) and neutral pH (pH 6.8). The plates were incubated at 28°C for 4–15 d (22, 45). The evaluation of the Al tolerance of isolates used the same scoring method as that for NaCl tolerance. Experiments were performed in triplicate.

### Symbiotic performance of isolates

Forty-four out of the 120 rhizobial isolates obtained were selected as representatives of diverse groups assessed by abiotic stress tolerance assays (Table S1). The symbiotic performance of the selected rhizobial strains was tested. Isolates were grown in YM broth at 28°C for 4–5 d as described by Vincent (74). Prior to the inoculation, soybean seeds were surface-sterilized with 70% (v/v) ethanol for 30 s, 3% (v/v) of sodium hypochlorite for 2 min, and then washed four times with sterile distilled water. Ten milliliters of the rhizobial cell suspension at 10^9^ cells mL^−1^ was then inoculated onto soybean seeds. Plants were grown aseptically in a growth chamber under the same conditions as those described for the preceding isolation experiment from pots. All treatments were performed in triplicate for each isolate, and non-inoculated plants served as the control (74). After 4 weeks, intact root nodules were assessed for nitrogen fixation activities using the acetylene reduction assay (ARA). A Shimadzu GC-2014 gas chromatograph (Shimadzu Corporation, Kyoto, Japan) equipped with a Porapak N column (Agilent Technologies, Santa Clara, CA, USA) was used for the assay; the incubation time for the assay was 30 min, and was followed by counting of the number of root nodules. To obtain accurate root nodule weights, root nodules were dried at 80°C for 48 h prior to weighing. Data were subjected to a statistical analysis by Tukey’s test using Statistica 12.0 software (StatSoft, Tulsa, OK, USA). The plant test was performed as described with strictly axenic and sterile (including UV irradiation) conditions using an EYELA FLI-2000 incubator (Tokyo Rikakikai Corporation, Tokyo, Japan).

### Isolation of genomic DNA

Forty-four out of 120 isolates were selected based on differences in their origins and stress tolerance levels (Table S1). Genomic DNA was extracted from isolates grown in YM broth medium at 28°C for 4 d. Prior to DNA isolation, cells were collected and washed twice with equal volumes of TE buffer. Total genomic DNA was extracted from isolates using 55 μL of 10% (v/v) cetyltrimethylammonium bromide, as described by Djedidi *et al*. (22). DNA concentrations and purities were assessed using a NanoDrop 2000 UV–vis spectrophotometer (Thermo Fisher Scientific, Wilmington, DE, USA).

### DNA amplification and sequencing

The PCR amplification and sequencing of the 16S rRNA, *nodD* (Nodulation D), and *nifH* (Nitrogenase iron protein) gene regions were performed using the method described by Risal *et al*. (60). The *recA* (DNA recombinase A), *atpD* (ATP synthase), and *glnA* (glutamate synthase) primers were described by Gaunt *et al*. (29) for α-rhizobia and by Baldwin *et al*. (4) for β-rhizobia. The *nifH* primer set described by Laguerre *et al*. (39) was used in the present study. Two distinct primer sets were used for the nodulation gene because the amplification of the *nodD* gene region using known primer sets is difficult in *Burkholderia* isolates. Therefore, in the case of α-rhizobia, we applied the primer set described by Risal *et al*. (60). Regarding isolates classified as β-rhizobia based on 16S rRNA sequences, a 380–395-bp fragment containing the intergenic sequence between the *nodD* and *nodB* genes was amplified and sequenced using the primers 5′-CAGATCNAGDCCBTTGAARCGCA-3′ (located at the 3′ end of *nodD* in rhizobia) and 5′-GGRTKNGGNCCRTCRTCR AANGT-3′ (located at the 5′ end of *nodB* in rhizobia), as described by Chen *et al*. (12). PCR amplification was performed using 50-μL reaction mixtures with the following composition: 2.5 μL primer sets (10 μM each), 0.5 μL *Taq* DNA polymerase (Ex Taq^TM^ polymerase, 5 U mL^−1^; Takara Bio, Ohtsu, Shiga, Japan), 5 μL of 10× reaction buffer, 4 μL of 2.5 mM dNTPs mixture, and 1 μL DNA template (200–250 ng DNA). Thermal cycling conditions were as follows: denaturation at 94°C for 5 min, then 30 cycles of denaturation at 94°C for 1 min, annealing at 55°C for 1 min, and extension at 72°C for 3 min, followed by final extension at 72°C for 7 min. Amplification was performed using a thermal cycler (GeneAmp PCR system 9700; Applied Biosystems, Waltham, MA, USA). PCR products were checked in a 1.5% (w/v) agarose gel with 0.5×TBE buffer mixed with 0.5 μg mL^−1^ ethidium bromide. A 1-kb DNA ladder was used as a marker. Regarding all of the genes amplified, the bands were subsequently excised and DNA was purified using the QIAEX^®^ II Agarose Gel Extraction Kit (QIAGEN, Hilden, Germany). PCR products were sequenced by Eurofins Genomics K.K. (Eurofins Nihon Kankyo, Tokyo, Japan; http://eurofinsgenomics.jp), in accordance with the manufacturer’s protocols and using the primers previously described. Multiple sequences were compared with those deposited in the GenBank database using the online BLAST algorithm-based sequence alignment. Phylogenetic trees were constructed based on a neighbor-joining analysis using Genetix 11 and MEGA 6.0 software. The analysis for 16S rRNA and housekeeping genes was based on multilocus sequence typing (MLST)

### Nucleotide sequence accession numbers

The sequences for the different genes obtained in the present study were deposited in the DNA Databank of Japan (DDBJ) under accession numbers LC104283 to LC104306 (16S rRNA), LC107279 to LC107322 (the *recA* region), LC407120–LC407140 and LC407366–LC407433 (*atpD* and *glnA*), LC107518 to LC107561 (the *nifH* gene), and LC107562 to LC107605 (the intergenic spacer *nodDB* and *nodD* genes).

## Results and Discussion

### Characterization of soil-sampling sites of Venezuela and soybean-nodulating bacteria isolation

Among the locations of the Venezuelan soils analyzed in the present study, Trujillo and Merida are located in the Venezuelan Andes (Fig. 1, Table 1). These two soils were classified as acidic and have pH values ranging between 3.6 and 4.5 in Trujillo and between 4.4 and 5.0 in Merida (11). Falcon is located in northwest Venezuela and has been characterized as an aridic soil, showing a high soil pH ranging between 8.0 and 10. Apure and Trujillo soils were previously shown to contain a high Al ion concentration (11). Amazonas and Caracas (DC) soils contain moderate concentrations of Al, which induce physiological disorders in crops (11). Guárico and Anzoátegui are located in savannas and these soils show nutrient deficiencies, such as nitrogen and phosphorus, in cultivation.

Based on differences in the ecosystem, temperature, and vegetation at soil-sampling sites, Falcon is located in an arid desert area subjected to high water stress and temperatures that range between 22 and 40°C (Table 1). Four soil-sampling sites at Guárico, Lara, Apure, and Anzoátegui were categorized as a savanna, floodplain zone, xerophilic ecosystem, and savanna near the coast, respectively (Fig. 1). These sites showed high temperatures throughout the year ranging between 25 and 30°C at Guárico, 10 and 34°C at Lara, 10 and 34°C at Apure, and 25 and 38°C at Anzoátegui (Table 1). Regarding soybean cultivation, the Aragua Valley produces many types of legumes belonging to the genera *Vigna*, *Cajanus*, *Phaseolus*, and *Glycine* with two types of cultivation systems: one is conventional farming with chemical fertilization, whereas the other is organic farming. Anzoátegui is also a soybean production area using organic fertilizer. The remaining regions do not have a history of soybean cultivation. Amazonas is located in the Guiana Highlands in a rainforest ecosystem and is a traditional production area for crops including cucumber, tomato, and coriander (Fig. 1, Table 1).

We compared two methods for the isolation of soybean rhizobia: the field collection of soybean root nodules at Aragua and Anzoátegui, and the use of soybean seeds as a trap host to obtain root nodules. Forty-two isolates were obtained from soybean root nodules collected from soybean fields at Aragua and Anzoátegui (Table 1). Concerning the collection of rhizobial isolates from Venezuelan soils, we used the soybean cultivars ‘INIA’ and ‘Enrei’ as trap hosts to assess the diversity of the host specificities of Venezuelan soybean rhizobia. Consequently, 36 isolates were obtained from the root nodules of ‘INIA’ and 42 from those of ‘Enrei’. ‘Enrei’ did not have rhizobial isolates in Falcon, Guárico, or Merida soil.

### Physiological characterization of soybean-nodulating bacteria under abiotic stress conditions

A summary of the physiological properties of the 120 isolates examined is shown in Table S1. Regarding growth rates, the isolates tested were classified into three groups: fast growers (1–3 d) represented 8% of the total, intermediate growers (4–6 d) comprised 79%, and slow growers (more than 6 d) accounted for 13% (15 isolates). Concerning phenotypic properties, isolates were distinguished by colony morphologies: three isolates were white-yellow (WY), seven were creamy (C), six were transparent (T), 23 were white-transparent (WT), and the remaining 81 were white (W).

Under exposure to 2% NaCl, the growth rate of 26 isolates (21.7% of the total) differed from that of the control (without NaCl stress). More than 40% of the isolates obtained from Caracas, Miranda, Anzoátegui, and Apure showed low growth rates under exposure to 2% NaCl (Table S1). Only four isolates, namely, VAF128 from Aragua, VAp210 and VAp116 from Apure, and VLa19 from Lara, showed good growth rates in the presence of 4% NaCl. Thirty-three isolates showed 30–60% more growth in medium containing 3% NaCl than that of the control. Two isolates obtained from Aridisol in Falcon, six from Vertisol in savannas (Guárico and Lara), and two from Ultisol (Andes) showed 30–60% more growth on medium supplemented with 4% NaCl than that of the control. Previous studies described salt-tolerant isolates of *Rhizobium*. Marquina *et al*. (45) reported *Rhizobium* strains isolated from the Venezuelan root nodules of *Phaseolus* and *Leucaena* that survived exposure to 2% NaCl. Thus, the present results are consistent with previous findings in that the root-nodulating bacteria isolated from several specific agroecosystems in Venezuela showed high salt tolerance abilities (45).

The Al effect on bacterial growth under different pH conditions is shown in Table S1. The Al tolerance of isolates was greater under an acidic pH than a neutral pH. Seven isolates did not proliferate in medium incorporating 0.1 mM Al^3+^ under an acidic pH. Only three isolates (VAm22 from Amazonas, VMi21 from Miranda, and VTr35 from Trujillo) showed good growth rates in medium containing 2 mM Al^3+^ under an acidic pH (Table S1). In the case of Trujillo located in the Andes, the soil pH ranges between 3.6 and 4.5, and more than 50% of the isolates obtained from Trujillo grew in medium containing 2 mM Al^3+^ at pH 4. These results showed a relationship between the intensities of Al tolerance under acidic and neutral pH conditions and soil origins (42).

### Phylogenetic relationships of Venezuelan soybean root nodule bacteria based on MLST analysis

**Fig. 2:**
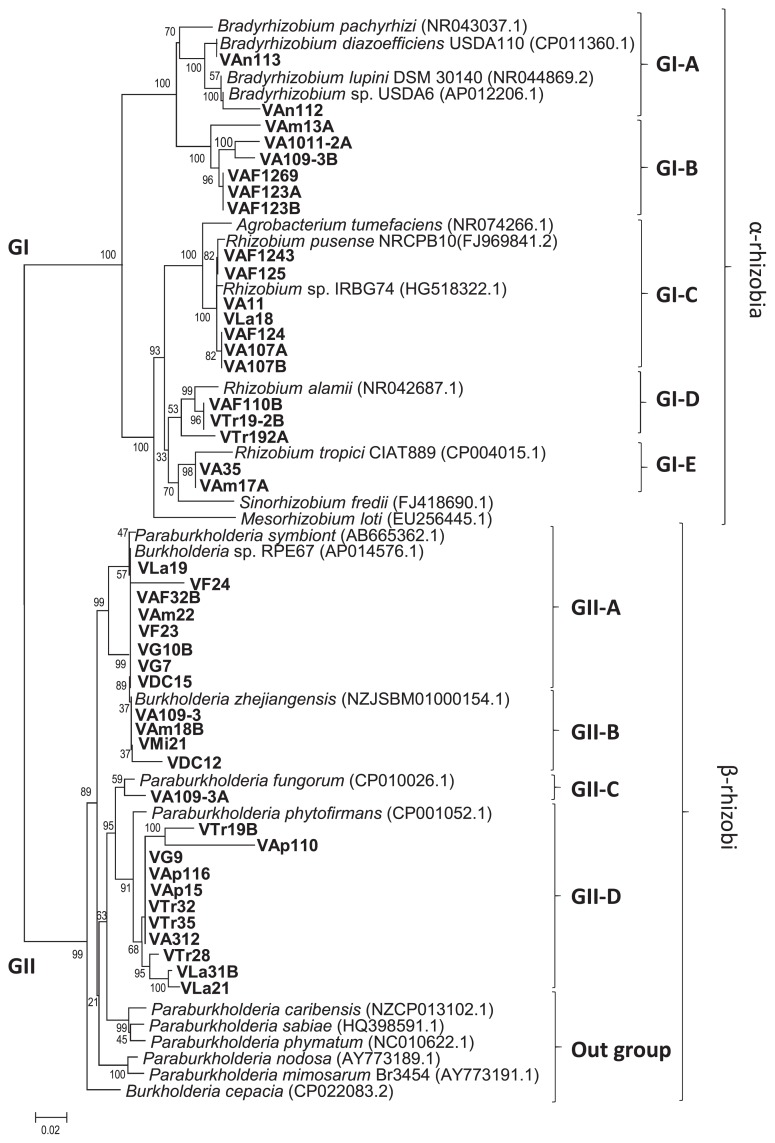
Phylogenetic tree based on MLST for 44 isolates of Venezuelan soybean-nodulating rhizobia and 22 reference strains representing six genera. The tree is based on 2,900-bp DNA fragments. Numbers at the nodes indicate the level of bootstrap support (%), based on a neighbor-joining analysis of 1,000 re-sampled datasets. The scale bar represents substitutions per nucleotide position with 0.02 changes per site. The isolate name included the elevation (MASL) and respective climatic zone of the sampling site.

Forty-four isolates were selected as representative isolates based on differences in responses when cultured under Al^3+^ and NaCl stress conditions (Table S1 and 1). A phylogenetic analysis with concatenated sequences (2,900 bp) for each isolate based on 16S rRNA gene (1,400 bp), *recA* region (550 bp), *atpD* (500 bp), and *glnA* (450 bp) sequences classified the 44 soybean isolates into two major groups designated as GI and GII (Fig. 2). Twenty isolates were included in the GI group with reference strains of the genera *Bradyrhizobium*, *Mesorhizobium*, *Rhizobium*, and *Sinorhizobium*. The remaining 24 isolates were placed in the GII group with reference strains of the genus *Burkholderia* (including the new reorganization as *Paraburkholderia*).

The GI group was divided into five subgroups. GI-A and GI-B included the five isolates, VA1011-2A, VA109-3B, VAF123A, VAF123B, and VAF1269, obtained from Aragua, VAm13A from Amazonas, and the two isolates VAn112 and VAn113 obtained from Anzoátegui were placed in the GI-A group with reference strains of the genus *Bradyrhizobium* (Fig. 2). These eight isolates showed a close relationship with *B. japonicum*, *B. lupini*, and *B. elkanii* (Fig. 2, Table 2 and 3). Rumjanek *et al*. (62) previously reported the distribution of *B. elkanii* in Brazil in 1993. Rodriguez-Navarro *et al*. (61) identified a novel *Bradyrhizobium* species, *B. pachyrhizi* that developed root nodules on members of the genus *Pachyrhizus* cultivated in several countries of the American continent in 2004. Regarding the distribution of *B. japonicum* in Central and South America (28), Koppell and Parker (37) showed the distribution of isolates related to *B. japonicum* in Panama in 2012. However, no information is currently available on the distribution of *B. lupini* in Central and South America, which makes this the first study for Venezuela. Furthermore, *Bradyrhizobium* was the predominant group in Anzoátegui soil.

The subsequent group included *Rhizobium*. This group was further divided into three subgroups designated GI-C, GI-D, and GI-E. The first subgroup (GI-C) including seven isolates, namely, VA11, VA107A, VA107B, VAF124, VAF1243, and VAF125 of Aragua, and VLa18 of Lara, showed a close relationship with *R. pusense*, *Rhizobium* sp. IRBG74, and *A. tumefaciens*. Pandy *et al*. (55) reported that *R. pusense* showed a close relationship with *R. rubi* and *A. tumefaciens* (*R. radiobacter*) and did not induce root nodules in chickpea or tumors in tobacco, which suggested that it is a non-nodulating, non-tumorigenic rhizobium (58, 59). In our analysis, seven isolates were classified into the GI-C clade with the reference strains of *R. pusense* and *Rhizobium* sp. IRBG74, a result that is consistent with previous findings (55, 59). Furthermore, *Rhizobium* sp. IRBG74 has been identified as a nodulating bacterium with a large plasmid. However, this is the first suggestion that a host plant develops root nodules in response to infection by *R. pusense*. In the GI-D clade, the isolates from Trujillo (VTr19-2B and VTr19A) and one from Aragua showed a close relationship with *R. alamii* (8, 78), a result that is consistent with the findings of the study by Berge *et al*. (8), which identified *Medicago ruthenica* and *M. sativa* (alfalfa) as host plants of *R. alamii* (73). Two isolates, VA35 (Aragua) and VAm17A (Amazonas), were categorized as relatives of *R. tropici* (32) (Fig. 2). The type strain of *R. tropici* CIAT899^T^ was isolated from common-bean root nodules in Colombia. The origin of *R. tropici* currently remains unclear. However, many strains have been isolated from *Phaseolus vulgaris* in a number of ecosystems in Brazil (27, 31, 32, 45. 47), Panama (37, 55), and Venezuela from *Leucaena* sp. (45). *Rhizobium* was the predominant group in Aragua soils with or without fertilization.

Twenty-four isolates classified in the GII clade were further grouped with *Burkholderia* or *Paraburkholderia* species (Fig. 2). The GII clade was divided into four subgroups and one outgroup. The first sub-group GII-A included eight isolates closely related to the *Paraburkholderia* symbiont and *Burkholderia* sp. RPE67, and these isolates are widely distributed in Venezuela, which is consistent with previous findings showing wide associations including the bean bug *Riptortus pedestris* (70). In addition, these isolates showed different stress tolerance levels, which may be enhanced by the capacity for fenitrothion degradation (70). Four isolates, namely, VAm18B (Amazonas), VA109-3 (Aragua), VMi21 (Miranda), and VDC12 (DC), were grouped into the GII-B subclade (Fig. 2). These isolates were closely related to *B. zhejiangensis*. However, there is currently no information on *B. zhejiangensis* as a root-nodulating bacterium or its distribution; it was previously identified as a methyl-parathion-degrading bacterium isolated from a wastewater treatment system in China (44). One isolate, VA109-3A isolated from Aragua (Inceptisol), was classified into the GII-C subclade with *Paraburkholderia fungorum*. Barrette *et al*. reported the coexistence of bacterial cells belonging to the genera *Burkholderia*, *Cupriavidus*, and *Rhizobium* in the root nodules of *M. pigra* and *M. pudica* in Costa Rica (6). The *Burkholderia* lineage including *P. fungorum* are root nodule symbionts for *M. pigra* (6). However, no information is available on the root nodulation ability of *P. fungorum* on soybean. De Oliveira-L *et al*. (21) showed that *P. fungorum* is commonly found in soil samples and promotes common bean growth in a dystrophic oxisol. *P. phytofirmans* is closely related to *P. fungorum* (95 % similarity) based on gene sequences (principally based on 16S rRNA, data not shown).

Eleven isolates were classified into the GII-D subclade and were closely related to *P. phytofirmans* (Fig. 2, Table 2). Sessitsch *et al*. (63) characterized the type strain PsJN^T^ of *P. phytofirmans* isolated from surface-sterilized onion roots grown in Dutch soil (63). This isolate was found to be a highly effective plant-beneficial bacterium and was able to establish rhizospheric and endophytic populations associated with a number of plants (76). However, its root nodulation ability remains unclear. Among the 11 soil-sampling sites, *P. phytofirmans* was present as a soybean root-nodulating bacterium at five (Table 3). At Apure and Trujillo, *P. phytofirmans* was considered to be the predominant soybean-nodulating bacteria (Table 3). The reference *Burkholderiaceae* species, *P. sabiae*, *P. caribiensis*, *P. phymatum*, *P. nodosa*, *P. mimosarum*, and *B. cepacia*, were grouped as an outgroup (Fig. 2). Several *Burkholderia* species have been reported as plant growth-promoting bacteria, which is consistent with the present results; *e.g*. the *P. sabiae* strain Br3407^T^ was inoculated onto *M. caesalpiniifolia* seedlings and, after 6 months of growth, produced very active root nodules that exhibited high acetylene reduction activities (13, 14, 25, 67). Regarding isolation and distribution, different (*Para*) *Burkholderia* species have been isolated in South America or from tropical countries (13, 46), such as the type strain PAS44^T^ (*P. mimosarum*), which was isolated from the root nodules of *Mimosa* in plants in South America (including Venezuela) and Taiwan (12, 13). However, these strains have not yet been demonstrated to function as soybean-nodulating bacteria. In the present study, the predominant isolate group was related to (*Para*) *Burkholderia*, with a wide distribution and dominance in floodplain (Apure) and savanna (Guárico) soils. The second dominant group was *Rhizobium*, with the distribution of α-rhizobia in soils depending on a history of legume cultivation.

Our phylogenetic analysis based on two (16S rRNA and RecA) genes suggested inaccuracies between α-rhizobia and β-rhizobia. However, MLST with four genes clarified the issues associated with previous identification (4). In contrast, Eisen (23) compared the topology of trees based only on the amino acid sequence of RecA and the nucleotide sequence of 16S rRNA from the same species. The present results suggested that β-rhizobia required specific gene translators to acquire phylogenetic interpretation (4). The nodulation process and molecular signaling currently remain unknown.

### Phylogenetic relationship of Venezuelan soybean-nodulating bacteria based on *nod* gene sequences

**Fig. 3:**
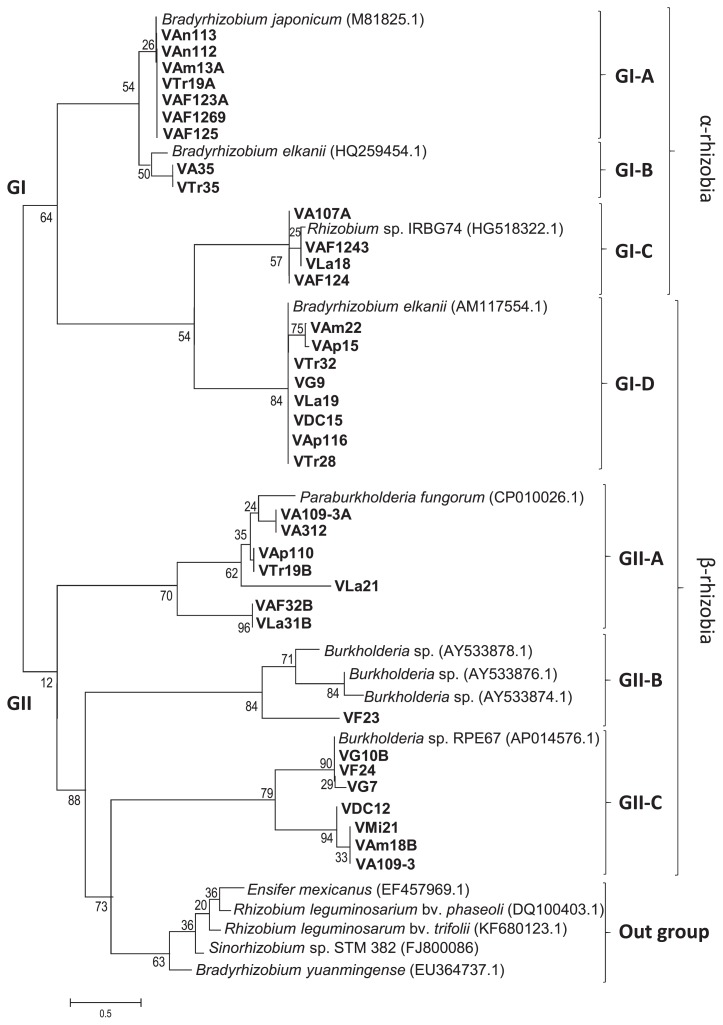
Phylogenetic tree based on intergenic sequences of *nodD*, nod box, and the *nodB* gene for 44 isolates of Venezuelan soybean-nodulating rhizobia and reference strains. Numbers at the nodes indicate the level of bootstrap support (%) based on 380–395-bp DNA fragments and a neighbor-joining analysis with 1,000 replications. The scale bar indicates 0.5 changes per site.

A phylogenetic analysis of *nodDB* intergenic sequences for Venezuelan isolates and 14 reference strains of *Bradyrhizobium*, *Burkholderia*, *Ensifer*, *Rhizobium*, and *Sinorhizobium* was performed. The isolates were classified into 2 groups, similar as MLST (Fig. 2): one group (GI) contained α-rhizobia (21 isolates, *Bradyrhizobium* and *Rhizobium*) and another group (GII) contained 15 Venezuelan isolates, *Burkholderia*, *Paraburkholderia*, and outgroup references strains (Fig. 3). Although 36 isolates were classified, the remaining isolates (eight) belonging to α-rhizobia isolates did not show bands with the primer for the intergenic spacer *nodDB*. Twelve isolates showed a single band and were divided into 2 subgroups. The isolates grouped in GI-A based on MLST analysis (Fig. 2) have the same position with *nodDB* (GI-A, Fig. 3), which had a close relationship with *Bradyrhizobium* species. For example, VAn112 and VAn113 (from Anzoátegui site) belonged to *B. lupini* and *B. diazoefficiens*, respectively. These isolates were closely related to *B. japonicum*. GI-A also contained 2 isolates that were classified as *R. alamii* (VTr19A) and *R. pusense* (VAF125). GI-B contained only 2 isolates: one from Aragua (VA35) classified as *R. tropici* and one from Trujillo (VTr35), which was classified as *P. phytofirmans*. Four isolates that were classified as *Rhizobium* sp. IRBG74 (based on MLST) were closely related to the same *Rhizobium* into GI-C. The last group of α-rhizobia, GI-D, contained only one reference strain *B. elkanii* and eight isolates of β-rhizobia (Fig. 3, Table 3). All these isolates were classified as β-rhizobia based on MLST (Fig. 2; three isolates as *P. symbiont*, GII-A, and five as *P. phytofirmans*, GII-D). These results strongly suggested horizontal gene transfer between (*Para*) *Burkholderia* and *B. elkanii*.

The remaining 15 isolates were classified as β-rhizobia into GII. The GII clade was further subdivided into the GII-A, GII-B, and GII-C subclades and one outgroup. Six isolates of *P. fungorum and P. phytofirmans* were classified as GII-A. This clade also included one isolate VAF32B of *Burkholderia* sp. Isolate VF23 at Falcon belonging to *Burkholderia* sp. was grouped in subclade GII-B with three reference strains (Br3461, MAP3-5, and Br3454 of *Burkholderia* sp.). These strains were previously reported by Chen *et al*. (13) to be symbionts of *M. bimucronata*, *M. pigra*, and *M. scabrella*, respectively. Seven isolates and a reference strain of *Burkholderia* sp. RPE67 were classified into subclade GII-C. Three isolates of VG7 and VG10B at Guárico and VF24 at Falcon showed close relationships with the same *Burkholderia* sp. RPE67. The other four isolates of VMi21 at Miranda, VA109-3 at Aragua, VAm18B at Amazonas, and VDC12 at DC of *B. zhejiangensis* were grouped into this clade at different clade branches due to 21% divergence. This group was related to *Burkholderia* sp. RPE67. Five strains of *Rhizobium*, *Sinorhizobium*, and *Bradyrhizobium* were classified into the subclade as an outgroup, which lacked Venezuelan isolates. Regarding relationships among species based on intergenic spacer *nodDB* sequences, *Burkholderia* or *Paraburkholderia* were more likely to harbor *nodD* genes transferred from *B. elkanii*. To verify this hypothesis, further analyses of the soybean root nodule bacteria of *Burkholderia* are needed. Amadou *et al*. (2) reported the genome sequence of the β-rhizobium *C. taiwanensis* and performed a comparative genomic analysis of rhizobia (2). Amadou *et al*. showed that *C. taiwanensis* carried 10 nodulation genes, *nodBCIJHASUQ*, one regulatory gene *nodD*, and a NodD-dependent regulatory consensus sequence (nod box) in a single 10-kb region (2). An analysis of the intergenic regions of the *nodD* and *nodB* gene sequences in the present study revealed that the isolates contained the intergenic region of nod box (10). Bournaud *et al*. (10) characterized *Burkholderia* species nodulating the Piptadenia Group (tribe *Mimoseae*) in Brazil using two neutral markers (16S rRNA and *recA* genes) and two symbiosis genes (*nodC* and *nifH* genes) to assess species affiliations and the evolution of symbiosis genes (10). Phylogenetic positions based on the 16S rRNA sequences of *Burkholderia* species nodulating the Piptadenia Group were strongly congruent with those based on *recA* sequences (10), and these findings were consistent with the present results. Thus, *Burkholderia* species nodulating in Brazil may have evolved mainly through vertical transfer, whereas (*Para*) *Burkholderia* species nodulating soybean may have evolved mainly through horizontal transfer. Further studies are needed to test this hypothesis.

**Fig. 4:**
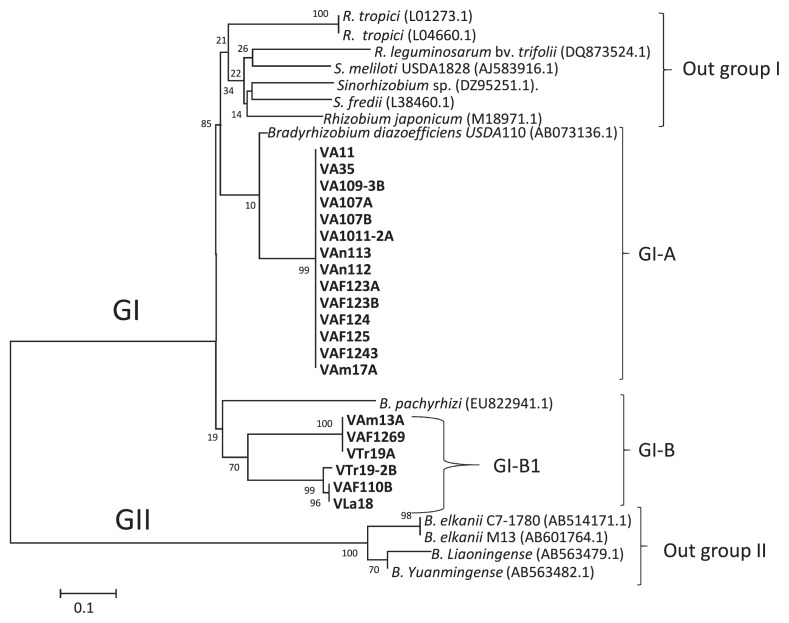
Phylogenetic analysis based on *nodD* gene region sequences of α-rhizobia isolates from Venezuela. The tree based on a 685-bp DNA fragment of the *nodD* gene obtained from Venezuelan *Bradyrhizobium* and *Rhizobium* isolates. The numbers at the branch nodes indicate bootstrap values (%) based on a neighbor-joining analysis of 1,000 re-sampled datasets. The scale bar indicates substitutions per site. (*B.: Bradyrhizobium*).

A phylogenetic tree was constructed based on *nodD* gene sequences for α-rhizobia (20 isolates of *Bradyrhizobium* and *Rhizobium*). Eight isolates belonging to α-rhizobia isolates did not show bands with the primer for the intergenic spacer *nodDB*. To clarify their positions, the *nodD* sequence was analyzed. All isolates showed a close phylogenetic relationship with *Bradyrhizobium* species (Fig. 4). Twenty isolates and 13 reference strains were classified into the GI and GII clades. Four strains of *Rhizobium* and four isolates of *Sinorhizobium* were classified as outgroup I, which lacked Venezuelan isolates. Fourteen Venezuelan isolates were classified into the GI-A subclade. These isolates showed a close relationship with the reference strain of *B. diazoefficiens* USDA110. These results included all α-rhizobia isolates classified into GI-A based on MLST, which showed a close relationship with *B. diazoefficiens* USDA110. Eleven of these isolates were obtained from Aragua (Fig. 4). Isolates that did not show bands with the intergenic spacer *nodDB* were classified as follows: VA1011-2A, VA109-3B, and VAF123B (based on MLST as the *Bradyrhizobium* clade) showed a close relationship with *B. diazoefficiens* USDA110 with the *nodD* gene (Fig. 4, Table 3). However, the isolates VA11 and VA107B of *Rhizobium* sp. IRBG74 and VAm17A of *R. tropici* were closely related to *B. diazoefficiens* USDA110 (Fig. 4, Table 3). The isolates of *R. alamii*, VTr19-2B at Trujillo, and VAF110B at Aragua were classified into subclade GI-B (Fig. 4, Table 3). The *nodD* sequences for these isolates showed a close relationship with the reference strain of *B. pachyrhizi*. Clade GII consisted of four reference strains of *B. elkanii*, *B. liaoningense*, and *B. yuanmingense* as outgroup II.

Sullivan and Ronson (68) previously reported that 500 kb of a symbiotic island of *Mesorhizobium* may be transferred from a symbiotic strain to a non-symbiotic soil bacterium in the field. *Bradyrhizobium* also contains a symbiotic island (35, 43, 52, 68). Venezuelan isolates showed similar results to the findings described by Barcellos *et al*. (5), namely, the horizontal transfer of symbiosis genes from *B. japonicum* to the indigenous *Ensifer fredii* and *B. elkanii* in Brazilian savanna soil (5). Furthermore, the present results suggest a high-affinity interaction between *Burkholderia* and *Bradyrhizobium* under Venezuelan conditions.

### Phylogenetic relationship of Venezuelan soybean-nodulating bacteria based on *nifH* gene sequences

**Fig. 5:**
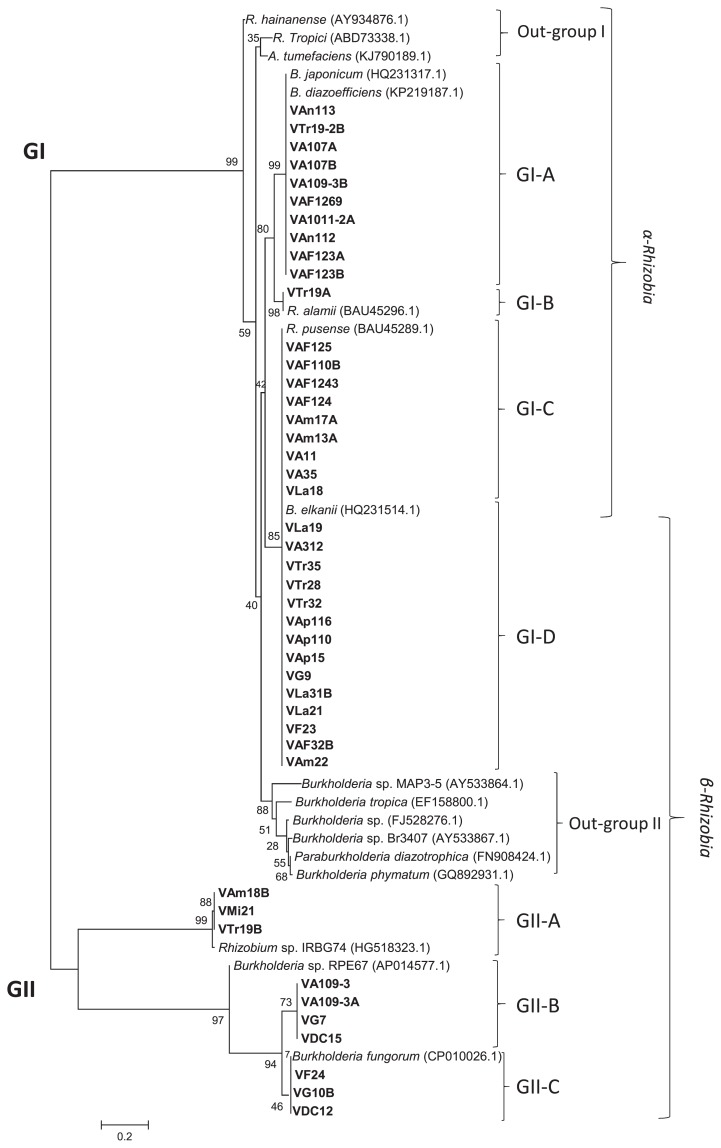
Phylogenetic tree based on *nifH* sequences for isolates of Venezuelan soybean-nodulating rhizobia. A 750-bp fragment of the *nifH* gene obtained from α-rhizobia and β-rhizobia isolates and reference strains was analyzed. The numbers at the branch nodes indicate bootstrap values (%) based on a neighbor-joining analysis of 1,000 re-sampled datasets. The scale bar indicates 0.2 changes per site. (*B.: Bradyrhizobium*).

A phylogenetic tree based on a 750-bp fragment from the *nifH* gene sequences of Venezuelan isolates and 17 reference strains is shown in Fig. 5. These isolates and reference strains were divided into two major clades designated as GI and GII. The GI clade contained 20 isolates of *Bradyrhizobium* and *Rhizobium*, 11 of *B. phytofirmans*, and three of *Burkholderia* sp. (Fig. 5, Table 3). The GI clade was further subdivided into the GI-A, GI-B, GI-C, and GI-D subclades. Three references strains, *R. hainanense*, *R. tropici*, and *A. tumefaciens*, were grouped without any Venezuelan isolates as outgroup I. The remaining 34 isolates tested were grouped into the next subclades. GI-A consisted of 11 α-rhizobia isolates belonging to *Bradyrhizobium*, and 8 of these isolates possessed the same position as *Bradyrhizobium* as previous phylogenetic trees, such as *nodD* (Fig. 4). One isolate (VTr19A) was placed in subclade GI-B and classified as *R. alamii*. Twenty-three isolates were placed in subclades GI-C and GI-D together with *R. pusense* and *B. elkanii* (65, 80). The isolates VAF125, VAF110B, VAF1243, VAF124, VAm17A, VAm13A, VA11, VA35, and VLa18 were grouped as six isolates of *R. pusense*, two of *R. tropici*, one of *R. alamii*, and one of *Bradyrhizobium* sp. based on MLST sequences (Fig. 5, GI-C). GI-D contained 14 isolates, VLa19, VA312, VTr35, VTr28, VTr32, VAp116, VAp110, VAp15, VG9, VLa31B, VLa21, VF23, VAF32B, and VAm22, belonging to the genus *Burkholderia* based on MLST sequences (10 isolates of *P. phytofirmans* and four of *Burkholderia* sp.). These results indicated that the *nifH* genes of these *Burkholderia* isolates were closely related to those of *R. pusense* and *B. elkanii*. The five reference strains of the genus *Burkholderia* were classified without any Venezuelan isolates (Outgroup II). *Burkholderia* sp. (FJ528276.1) was previously reported to be the predominant nodulating and nitrogen-fixing symbiont of legume nodules in a Brazilian pine forest (27, 40). *B. diazotrophica* is a symbiont of *M. pudica* isolated from French Guiana by Mishra *et al*. (50). *B. phymatum* is diazotrophic in novel plant-associated *Burkholderia* species (44, 72). The present results indicate that the *nifH* gene in *Burkholderia* in subclade GI-D is more closely related to the *nifH* genes of soybean rhizobia than to those of *Mimosa* symbionts.

The GII clade contained the remaining 10 isolates of *Burkholderia* and 3 references strains. This clade was divided into three subclades, designated as GII-A, GII-B, and GII-C (Fig. 5). The isolates VAm18B, VMi21, and VTr19B, shown to belong to *Burkholderia* based on MLST sequences, were classified into subclade GII-A with the reference strain *Rhizobium* sp. IRBG74. *Rhizobium* sp. IRBG74 is the first known nitrogen-fixing symbiont in the aquatic legume *Sesbania* sp. and is also a growth-promoting endophyte of wetland rice (16). Seven *Burkholderia* isolates, VA109-3, VA109-3A, VG7, VDC15, VF24, VG10B, and VDC12, were grouped into subclades GII-B and GII-C with the reference strains *Burkholderia* sp. RPE67 and *P. fungorum*. *Burkholderia* sp. RPE67 was previously reported to be a bacterial gut symbiont of the bean bug *R. pedestris* (36, 70, 71). *P. fungorum* was isolated as a relative of pathogenic *Burkholderia* (67). However, it is currently classified as a beneficial bacterium and its complete genome sequence has been published.

### Relationships among sampling sites and the distribution of Venezuelan soybean-nodulating bacteria

The distribution of Venezuelan soybean-nodulating bacteria is shown in Table 2 and 4. Among the 11 different ecosystem sites, comprising six sites at Aragua with and without fertilized fields, Amazonas in rainforests, Anzoátegui and Lara in savannas, and Trujillo in the Andean mountains showed the presence of *Rhizobium* and *Bradyrhizobium* species. Only two isolates of the genus *Bradyrhizobium* were isolated at Anzoátegui in savannas. Woomer *et al*. (77) analyzed the ecological indicators of native rhizobia in the tropical soils of Inceptisols, Mollisols, Ultisols, and an Oxisol on the island of Maui. The findings obtained showed that the most frequently occurring rhizobia were *Bradyrhizobium* species, which were present at 13 out of the 14 sites tested with a maximum of 10^5^ cells g^−1^ soil. In Venezuelan soils from the 6 sites, with the exception of Lara (Vertisol), the soil types of the sites were categorized as Inceptisol, Oxisol, and Ultisol, respectively, and *Bradyrhizobium* species were isolated from these soils, with the exceptions of Vertisol at Lara and Ultisol at Trujillo. Damirgi *et al*. (19) previously reported different distributions of *B. japonicum* serogroups 123 and 135 in relation to soil pH. Serogroup 123 was mainly distributed in soil with pH 5.9, whereas serogroup 135 was mainly distributed in soil with pH 8.3 (19). Previous studies demonstrated the influence of acidity on root nodulation (3, 26, 64). Martins *et al*. (48) reported the growth characteristics and symbiotic efficiency of rhizobia isolated from cowpea nodules in the northeast region of Brazil. The findings obtained showed that *Bradyrhizobium* was the predominant group of cowpea rhizobia, which grew well in alkaline media, whereas *Rhizobium* exhibited good growth in acidic media (48). In the present study, *Bradyrhizobium* was the predominant nodulating bacteria at Anzoátegui, at which the soil pH ranges between 8.0 and 10.0, whereas *R. alamii* was the predominant nodulating bacteria at Trujillo at a soil pH range of between 3.6 and 4.5. These results are consistent with previous findings.

Ten out of the 11 different ecosystem sites, with the exception of Anzoátegui, showed the presence of soybean root nodule bacteria classifiable into the genus (*Para*) *Burkholderia*. All soybean root nodule bacteria obtained from the five sites of Apure in floodplains, Caracas in national parks, Miranda on mountain slopes, Falcon in arid deserts, and Guárico in savannas were members of (*Para*) *Burkholderia*. Soil types at the five sites were classified as Inceptisol, Alfisol, Aridisol, and Vertisol (Table 4).

In the case of Aridisol of Falcon and Alfisol of Caracas and Miranda, all soybean nodule bacteria isolated were phylogenetically classified into the genus *Burkholderia*. Alfisol generally develops under forest vegetation, whereas Aridisol occurs in a dry climate and often contains the accumulation of salts, gypsum, or carbonates (11, 54). Differences in distribution among *Rhizobium*/*Bradyrhizobium* and (*Para*) *Burkholderia* species in Alfisol and Aridisol may be attributed to the forest vegetation and desert ecosystems, respectively.

Stopnisek *et al*. (66) analyzed the biogeographical distribution of soil *Burkholderia* populations and indicated that *Burkholderia* species have high competitiveness in acidic soils, but are outcompeted in alkaline soils. The findings obtained also revealed that pH tolerance is a general phenotypic trait of the genus *Burkholderia*. In the present study, three Inceptisol of Aragua with and without fertilized fields and Apure were used as rhizobial isolation sources. At Aragua, soil pH ranged between 7.1 and 8.0; therefore, (*Para*) *Burkholderia* species were not the predominant soybean root nodule bacteria. At Apure, *Paraburkholderia* was the predominant genus of soybean root nodule bacteria (Table 2). This difference may reflect the low soil pH at Apure and the high competitiveness of the genus (*Para*) *Burkholderia* in acidic soils, as reported by Stopnisek *et al*. (66).

Fourteen out of the 24 isolates (58.3%) classified into the genus *Burkholderia* survive on media containing 1 mM Al^3+^ or a higher concentration at pH 4.5, whereas only five isolates (25.0%) of 20 isolates classified into *Rhizobium*/*Bradyrhizobium* survive under the same conditions. Kunito *et al*. (38) showed that the genera *Burkholderia* and *Lipomyces* were the predominant Al-resistant microorganisms isolated from acidic Inceptisol soil. This finding is consistent with the present results.

Eight out of the 11 sites showed the presence of soybean root nodule bacteria classified as *P. fungorum*. *P. fungorum* was the predominant soybean root nodule bacteria in Inceptisol at Apure and in Ultisol at Trujillo under low soil pH. The species *B. zhejiangensis* and *P. phytofirmans* were present in Inceptisol, Oxisol, Alfisol, and Vertisol soils at neutral pH. These results indicate that the distribution of soybean root nodule bacteria classified as *Rhizobium* and *Bradyrhizobium* was regulated by soil pH and type (79). However, the distribution of (*Para*) *Burkholderia* was presumed to be influenced less by soil pH and type.

*P. fungorum* was isolated as a fungal endosymbiont from *Phanerochaete chrysosporium*, and shows the ability to degrade aromatic compounds (21). Ferreira *et al*. (27) initially reported that *P. fungorum* was a root-nodulating bacteria that was isolated from Amazonian soil (Brazil) using *P. vulgaris* as the trap host. Previous studies demonstrated the occurrence of root-nodulating bacteria of the β-rhizobia type as symbionts, particularly in *Mimosa* species, which have been isolated in Costa Rica, Texas (6, 25), Panama (56), Brazil, Venezuela (9, 12, 28), Taiwan (12, 13), India, Papua New Guinea (15, 25), Australia, China (41), and French Guiana (50). However, *P. fungorum* and *B. zhejiangensis* did not form root nodules on soybean. In the present study, *P. fungorum* and *B. zhejiangensis* were widely distributed across the climatic regions and wide soil pH ranges of Venezuela (Table 2 and 4).

### Symbiotic performance of Venezuelan soybean-nodulating bacteria

The symbiotic performance of isolates belonging to the different phylogenetic groups based on MLST of 16S rRNA, *recA*, *atpD* and *glnA*, *nifH*, and *nodD* gene sequences is shown in Table 3.

The root nodule number of isolates belonging to the genera *Rhizobium* and *Bradyrhizobium* ranged between 18±0.5 and 50±1.5. The ARA of isolates belonging to these two genera ranged between 24.0±1.0 and 83.6±1.0 nM C_2_H_4_^−1^ h^−1^ g^−1^ dry weight of root nodules. VAm17A isolated from Oxisol at Amazonas showed the highest root nodule number (50±1.5) for *Rhizobium*/*Bradyrhizobium* isolates (Fig. 2, Table 3). Isolates classified as *R. alamii* exhibited the capacity for nodulation and nitrogen-fixation activity; however, the nodulation ability of *R. alamii* currently remains unclear. Regarding α-rhizobia, VTr19-2B classified as *R. alamii* displayed the highest ARA of 83.6±1.0 nM C_2_H_4_^−1^ h^−1^ g^−1^ dry weight of nodules. In contrast, VAF123A and VAF123B showed the lowest nodulation activities as well as the lowest ARA (Table 3). A phylogenetic analysis of *nifH* gene sequences indicated that some isolates of *R. pusense* and *R. tropici* were closely related to *R. pusense*. These isolates also showed high ARA activities that ranged between 52.1 and 78.5 nM C_2_H_4_^−1^ h^−1^ g^−1^ dry weight of nodules.

The ARA activities of isolates belonging to the genus (*Para*) *Burkholderia* ranged between 23.5±3.0 and 86.6±2.5 nM C_2_H_4_^−1^ h^−1^ g^−1^ dry weight of nodules. Five isolates consisting of VAp116 and VAp15 at Apure and VTr19B, VTr32, and VTr35 at Trujillo were classified into the genus (*Para*) *Burkholderia* and displayed higher root nodule numbers than α-rhizobia. The *nodD* gene sequences of these isolates showed a close relationship with those of *B. elkanii* and *Burkholderia* sp. (Table 3). The VTr35 isolate showed the highest ARA activity at 86.6±2.5 nM C_2_H_4_^−1^ h^−1^ g^−1^ dry weight of nodules among all isolates tested. VTr35 was classified as *P. phytofirmans* (Fig. 2) and, in symbiotic genes, was related to *B. elkanii* (Fig. 3 and 5). Horizontal gene transfer has been reported under tropical conditions, which is consistent with the present results. For example, Sullivan and Ronson (68) reported symbiotic gene transfer from *R. loti* to other soil bacteria. Horizontal or lateral gene transfer was known to occur among prokaryotes for many years and plays a major role in prokaryote genome evolution (5, 9, 13, 43). In the case of soybean root nodule bacteria belonging to (*Para*) *Burkholderia*, horizontal gene transfer is suggested for nodulation and nitrogen fixation genes, which might occur between *B. elkanii* and *P. phytofirmans*. This assumption could explain the highest symbiotic potential of these isolates (Table 3). However, further studies are needed to confirm these results.

In the present study, we demonstrated that the genus (*Para*) *Burkholderia* was the predominant soybean root nodule bacteria in Venezuelan soils located in different climatic and topographical regions. In comparisons of symbiotic performance among isolates grouped into *Rhizobium*, *Bradyrhizobium*, and (*Para*) *Burkholderia*, the VAp15 isolate from Inceptisol at Apure and the VTr35 isolate from Ultisol at Trujillo belonging to the genus *Paraburkholderia* induced the highest root nodule numbers among the 44 isolates tested. VTr35 exhibited the highest nitrogen-fixation ability in root nodules. Furthermore, VTr35 showed the highest Al^3+^ tolerance and survived exposure to 2 mM Al^3+^ at pH 4.5. Thus, soybean root nodule bacteria classifiable as *Burkholderia*, such as the VTr35 isolate, may serve as a novel inoculant for soybean under abiotic stress conditions. However, further studies are needed to confirm the effectiveness of VTr35 as an inoculant for soybean under field conditions.

## Supplementary Information



## Figures and Tables

**Table 1:** General characteristics of soil-sampling sites and Venezuelan isolate numbers

Origin	Soil-sampling sites	Soil type	pH*	Temp. (°C)**	Non-legume vegetation of sampling sites	Total isolates on *G. max*		Representative isolates for phylogenetic analyses	
	
						‘INIA’	‘Enrei’	‘INIA’	‘Enrei’
**Soils (Inoculation test)**	**Amazonas**	Oxisol	5.5~6.0	12~33	Cucumber, tomato, coriander, *Capsicum* sp.	4	6	3	1
	**Apure**	Inceptisol	4.2~5.0	10~34	*Acacia* sp., *Caraipa* sp., several trees	6	3	3	0
	**Aragua**	Inceptisol	7.1~7.5	18~31	*Sesamun* sp.	6	6	2	1
	**Aragua**	Inceptisol	7.5~8.0		*Asteracea* sp., grasses	6	7	0	2
	**DC (Caracas)**	Alfisol	5.6~6.5	10~31	*Coffea* sp., sugarcane, tree forest, *Bryophytes*	3	4	0	2
	**Falcon**	Aridisol	8.0~10	22~40	Mining and *Prosopis* sp., *Opuntia* sp.	2	0	2	0
	**Guárico**	Vertisol	5.8~6.5	25~35	Species of grasses	4	0	3	0
	**Lara**	Vertisol	6.0~6.3	10~34	*Coffea* sp., *Inga* sp., grasses.	0	9	0	4
	**Merida**	Ultisol	4.4~5.0	−6~24	*Musa* sp., *Lactuca* sp., forestal species, *Theobroma*	1	0	1	0
	**Miranda**	Alfisol	5.6~6.0	10~33	Diverse species of tree, humid forest	1	1	0	1
	**Trujillo**	Ultisol	3.6~4.5	10~24	*Coffea* sp.	3	6	3	3

					Total number of isolates	36	42	17	14

**Field collection of root nodules**	**Aragua**	Inceptisol	7.1~7.5	18~31	Species of grasses	26	—	6	—
	**Aragua**	Inceptisol	7.5~8.0		*Asteracea* plants	9	—	5	—
	**Anzoátegui**	Ultisol	8~10	25~38	Avocado	7	—	2	—

					Total number of isolates	42		13	

* These values were reported by Casanova (11) and confirmed with the standard pH-soil method (11, 34)

** temperature average reported by REDBC and INIA-Venezuela.

**Table 2:** Relationships between Venezuelan isolates of soybean-nodulating bacteria and sampling sites, soil types, and tolerance levels of isolates to Al^3+^ 0.1–2 mM under acid conditions and 0–4% NaCl.

Site (Ecosystem)^a^	Soil type (pH range)	Strain Code	Deduced genus and species based on 16S rRNA	Al^3+^mM at pH 4.5 showing good growth	NaCl % showing good growth
Aragua (V-WF)	Inceptisol (7.1~7.5)	**VA11**	*R. pusense*	0.5	2
		**VA35**	*R. tropici*	0.5	3
		**VA1011-2A**	*B. japonicum*	1	2
		**VA107A**	*R. pusense*	0.1	3
		**VA107B**	*R. pusense*	0.1	3
		**VA109-3B**	*B. japonicum*	0.5	3

		**VA312**	*P. fungorum	0.1	0
		**VA109-3**	*B. zhejiangensis	1	4
		**VA109-3A**	*B. zhejiangensis	1	3

Aragua (V-F)	Inceptisol (7.5~8.0)	**VAF110B**	*R. alamii*	0.5	3
		**VAF123A**	*B. lupini*	0.5	4
		**VAF123B**	*B. lupini*	0	3
		**VAF124**	*R. pusense*	0.1	2
		**VAF1243**	*R. pusense*	1	3
		**VAF125**	*R. pusense*	1	4
		**VAF1269**	*B. lupini*	0.1	3

		**VAF32B**	*P. fungorum*	0.1	2

Amazonas (Am-r)	Oxisol (5.5~6.0)	**VAm17A**	*R. tropici*	1	2
		**VAm13A**	*B. elkanii*	0.5	3

		**VAm18B**	*B. zhejiangensis	1	3
		**VAm22**	*P. fungorum*	2	4

Anzoategui (S-NC)	Ultisol (8.0~10.0)	**VAn112**	*B. lupini*	0.1	0
		**VAn113**	*B. japonicum*	0.1	0

Apure (F)	Inceptisol (4.2~5.0)	**VAp110**	*B. fungorum	0.1	1
		**VAp116**	*B. fungorum	0.1	4
		**VAp15**	*B. fungorum	0.1	4

DC (N.P-C)	Alfisol (5.6~6.5)	**VDC12**	*Burkholderia sp.	1	4
		**VDC15**	*B. zhejiangensis	1	1

Falcon (A-D)	Aridisol (8.0~10.0)	**VF23**	*Burkholderia sp.	1	4
		**VF24**	*P. fungorum*	0.1	4

Guarico (S)	Vertisol (5.8~6.5)	**VG10B**	*Burkholderia sp.	1	4
		**VG7**	*Burkholderia sp.	1	4
		**VG9**	*P. fungorum*	0.1	4

Lara (X-NS)	Vertisol (6.0~6.3)	**VLa18**	*R. pusense*	0.1	4

		**VLa19**	*P. fungorum*	1	4
		**VLa21**	*P. phytofirmans*	0.1	2
		**VLa31B**	*P. fungorum*	1	4

Miranda (M)	Alfisol (5.6~6.0)	**VMi21**	*Burkholderia sp.	2	3

Trujillo (A)	Ultisol (3.6~4.5)	**VTr19-2B**	*R. alamii*	1	2
		**VTr19A**	*R. alamii*	0.1	1

		**VTr19B**	*P fungorum*	0.1	3
		**VTr28**	*P. fungorum*	2	3
		**VTr32**	*P. fungorum*	0.1	4
		**VTr35**	*P. fungorum*	2	4

* B.: Burkholderia. P.: Paraburkholderia. B.: Bradyrhizobium. R.: Rhizobium.

V-F: valley with fertilizer; S-NC: Xerophilic ecosystem near the coast; X-NS: xerophilic ecosystem nearest the savanna; V-WF: valley without fertilizer; Am-r: Amazon-rainforest; A-D: arid-like desert; A: Andes. N.P-C: National Park inside the city

**Table 3:** Summary of symbiotic performance of Venezuelan isolates of soybean-nodulating bacteria

Strain Code	Site (Ecosystem)^a^	Soil type	MLST analysis	*nod* genes^b^	*nifH*	Root nodule numbers^c^	ARA^d^
**VA11**	Aragua (V-WF)	Inceptisol	*R. pusense*	*B. japonicum*	*R. pusense*	21±3.0	64.1±1.5
**VA35**			*R. tropici*	*B. diazoefficiens*	*R. pusense*	48±3.1	68.4±2.0
**VA1011-2A**			*Bradyrhizobium* sp.	*Bradyrhizobium* sp.	*B. japonicum*	35±1.6	25.8±4.3
**VA107A**			*R. pusense*	*B. japonicum*	*B. japonicum*	24±6.0	44.2±5.0
**VA107B**			*R. pusense*	*B. japonicum*	*B. japonicum*	20±1.5	44±4.4
**VA109-3B**			*Bradyrhizobium* sp.	*Bradyrhizobium* sp.	*B. japonicum*	41±4.4	40.2±4.0
	
**VA312**			*P. phytofirmans*	*P. fungorum*	*B. elkanii*	24.7±4.0	51.3±3.0
**VA109-3**			*B. zhejiangensis	*Burkholderia sp.	*Burkholderia sp.	41±8.6	40.2±5.1
**VA109-3A**			*P. fungorum*	*P. fungorum*.	*Burkholderia sp.	42±7.0	23.5±3.0

**VAF110B**	Aragua (V-F)	Inceptisol	*R. alamii*	*Bradyrhizobium* sp.	*Bradyrhizobium* sp.	22±2.8	24±1.0
**VAF123A**			*Bradyrhizobium* sp.	*Bradyrhizobium* sp.	*B. japonicum*	17.6±3.0	25.3±1.3
**VAF123B**			*Bradyrhizobium* sp.	*Bradyrhizobium* sp.	*B. japonicum*	19±4.0	26±1.0
**VAF124**			*R. pusense*	*B. diazoefficiens*	*R. pusense*	38±1.7	52.1±4.0
**VAF1243**			*R. pusense*	*B. diazoefficiens*	*R. pusense*	34±3.7	31.4±4.2
**VAF125**			*R. pusense*	*B. diazoefficiens*	*R. pusense*	18±0.5	66.4±2.9
**VAF1269**			*Bradyrhizobium* sp.	*Bradyrhizobium* sp.	*B. japonicum*	22±1.5	49.4±1.8
	
**VAF32B**			*Burkholderia sp.	*Burkholderia sp.	*B. elkanii*	42.7±5.5	55.8±0.5

**VAm17A**	Amazonas (Am-r)	Oxisol	*R. tropici*	B*radyrhizobium* sp.	*R. pusense*	50±1.5	**78.5±1.2**
**VAm13A**			*Bradyrhizobium* sp.	*Bradyrhizobium* sp.	*R. pusense*	40±1.0	57.2±0.24
	
**VAm18B**			*B. zhejiangensis	*Burkholderia sp.	*Rhizobium* sp.	45±4.5	**77.6±1.2**
**VAm22**			*Burkholderia sp.	*B. elkanii*	*B. elkanii*	30±2.9	42.9±0.8

**VAn112**	Anzoategui (S-NC)	Ultisol	*B. lupini*	*B. diazoefficiens*	*B. diazoefficiens*	23.3±1.5	50.5±0.9
**VAn113**			*B. diazoefficiens*	*B. diazoefficiens*	*B. diazoefficiens*	22±1.8	43.2±3.9

**VAp110**	Apure (F)	Inceptisol	*P. phytofirmans*	*P. fungorum*	*B. elkanii*	35±1.5	58.3±4.0
**VAp116**			*P. phytofirmans*	*B. elkanii*	*B. elkanii*	58±3.1	69.9±2.0
**VAp15**			*P. phytofirmans*	*B. elkanii*	*B. elkanii*	**70±5.9**	**74.7±0.7**

**VDC12**	DC (N.P-C)	Alfisol	*B. zhejiangensis	*Burkholderia sp.	*Burkholderia sp.	17±1.5	26±0.3
**VDC15**			*Burkholderia sp.	*B. elkanii*	*Burkholderia sp.	25±3.2	32±1.9

**VF23**	Falcon (A-D)	Aridisol	*Burkholderia sp.	*Burkholderia sp.	*B. elkanii*	47.8±4.3	53.3±1.4
**VF24**			*Burkholderia sp.	*Burkholderia sp.	*Burkholderia sp.	46.4±2.1	45±1.0

**VG10B**	Guarico (S)	Vertisol	*Burkholderia sp.	*Burkholderia sp.	*Burkholderia sp.	38±2.5	57.1±2.0
**VG7**			*Burkholderia sp.	*Burkholderia sp.	*Burkholderia sp.	25.3±2.5	32±1.1
**VG9**			*P. phytofirmans*	*B. elkanii*	*B. elkanii*	35±3.3	44.4±0.9

**VLa18**	Lara (X-NS)	Vertisol	*R. pusense*	*Bradyrhizobium* sp.	*B. elkanii*	28.7±1.4	53.3±2.1
	
**VLa19**			*Burkholderia sp.	*B. elkanii*	*B. elkanii*	21±1.5	64±1.9
**VLa21**			*P. phytofirmans*	*Burkholderia sp.	*B. elkanii*	26±1.9	34±3.0
**VLa31B**			*P. phytofirmans*	*Burkholderia sp.	*B. elkanii*	33±5.0	51±1.4

**VMi21**	Miranda (M)	Alfisol	*Burkholderia sp.	*Burkholderia sp.	*Rhizobium* sp.	29±2.2	40±2.1

**VTr19-2B**	Trujillo (A)	Ultisol	*R. alamii*	*Bradyrhizobium* sp.	*B. japonicum*	35±1.5	**83.6±1.0**
**VTr19A**			*R. alamii*	*Bradyrhizobium* sp.	*R. alamii*	30±2.5	51.19±1.9
	
**VTr19B**			*P. phytofirmans*	*Burkholderia sp.	*Rhizobium* sp.	55.3±4.9	43.8±2.7
**VTr28**			*P. phytofirmans*	*B. elkanii*	*B. elkanii*	25±2.0	34.5±3.0
**VTr32**			*P. phytofirmans*	*B. elkanii*	*B. elkanii*	58±5.0	62.9±4.1
**VTr35**			*P. phytofirmans*	*B. elkanii*	*B. elkanii*	**68±6.8**	**86.6±2.5**

Sequences were compared with Blast in GenBank. MLST: multilocus sequence typing analysis (See the description of primers). *B.: Bradyrhizobium*.

* B.: Burkholderia

a V-F: valley with fertilizer; S-NC: Xerophilic ecosystem near the coast; X-NS: xerophilic near the savanna; V-WF: valley without fertilizer; Am-r: Amazon-rainforest; A-D: arid-like desert; A: Andes. N.P-C: National Park inside the city.

b Results are based on sequences for *Alphaproteobacteria* with the *nodD* gene and intergenic spacer *nodDB* on *Betaproteobacteria* (See the description of primers for nodulation)

c Results are the numbers of nodules per plant 4 weeks after the inoculation (mean standard deviations; *n*=3). Control plants (non-inoculated) had no nodules. The plant test was performed with *G. max* cultivar INIA

d Acetylene reduction assay (ARA). Values represent activity expressed as nmol C_2_H_4_^−1^ h^−1^ g^−1^ dry weight of nodules.

**Table 4:** Frequency of α- and β-rhizobia isolated from root nodules of soybean at different ecosystems in Venezuela.

Soil-sampling sites	Ecosystem	Soil type	Legume history	Frequencies (%) of α- and β-rhizobia in different soils	

				α-rhizobia	β-rhizobia^**^
**Amazonas**	Rainforest	Oxisol	*Phaseolus* sp., Fabaceae^*^	4.5^a^	4.5
**Apure**	Floodplain	Inceptisol	Fabaceae^*^	0	6.8
**Aragua**	Valley, no fertilizer	Inceptisol	*Vigna* sp., *Canajus* sp., *Arachis* sp.	13.6^a^,^b^	6.8
**Aragua**	Valley with fertilizer	Inceptisol	*Phaseolus* sp., *G. max*	15.9^a^,^b^	2.3
**DC (Caracas)**	National Park in the city	Alfisol	Fabaceae^*^	0	4.5
**Falcon**	Arid desert	Aridisol	Fabaceae^*^	0	4.5
**Guárico**	Savanna	Vertisol	—	0	6.8
**Lara**	Xerophilic ecosystem	Vertisol	*Phaseolus* sp.	2.3^b^	6.8
**Miranda**	Mountain	Alfisol	Fabaceae^*^	0	2.3
**Trujillo**	Andes	Ultisol	Fabaceae^*^	4.5^b^	9.1

**Anzoátegui**	Savanna near the coast	Ultisol	*G. max*	4.5^a^	0

			Total % of frequencies	45.3	54.4

Fabaceae^*^: It includes unknown genera of trees, shrubs, and perennial or annual herbaceous plants.

a It was classifiedas the genus *Bradyrhizobium*.

b it was related to the genus *Rhizobium*.

β-rhizobia^**^: all isolates related to the genus *Burkholderia* or *Paraburkholderia*.
